# Estimating the indirect prevalence of female genital mutilation/cutting in Switzerland

**DOI:** 10.1186/s12889-021-10875-w

**Published:** 2021-05-29

**Authors:** S. Cottler-Casanova, J. Abdulcadir

**Affiliations:** 1 Division of Gynaecology, Department of Paediatrics, Gynaecology and Obstetrics, Geneva University Hospitals, Boulevard de la Cluse 30, Geneva, 1211 Switzerland; 2 Department of Epidemiology and Public Health, Swiss Tropical and Public Health Institute, Basel, Switzerland; 3 University of Basel, Basel, Switzerland

**Keywords:** Female genital mutilation, Female genital cutting, Female genital mutilation/cutting, Indirect estimates, Prevalence, Switzerland

## Abstract

**Background:**

We updated the indirect estimates for women and girls living with Female Genital Mutilation Cutting (FGM/C) in Switzerland, using data from the Swiss Federal Statistical Office of migrant women and girls born in one of the 30 high-prevalence FGM/C countries that are currently living in Switzerland.

**Methods:**

We used Yoder and Van Baelen’s “Extrapolation of FGM/C Countries’ Prevalence Data” method, where we applied DHS and MICS prevalence figures from the 30 countries where FGM/C is practiced, and applied them to the immigrant women and girls living in Switzerland from the same 30 countries.

**Results:**

In 2010, the estimated number of women and girls living with or at risk of FGM/C in Switzerland was 9059, whereas in 2018, we estimated that 21,706 women and girls were living with or at risk of FGM/C.

**Conclusion:**

Over the past decade, there have been significant increases in the number of estimated women and girls living with or at risk of FGM/C in Switzerland due to the increase in the total number of women and girls originally coming form the countries where the practice of FGM/C is traditional.

**Supplementary Information:**

The online version contains supplementary material available at 10.1186/s12889-021-10875-w.

## Background

The practice of Female Genital Mutilation/Cutting (FGM/C) has been recorded in more than 31 countries around the world. The prevalence of the traditional practice has been widely documented, with standardized survey methodologies developed and refined over the past decades. The main surveys used to estimate prevalence of FGM/C are the Demographic Health Survey (DHS) developed by ICF International [1] and the Multiple Indicator Cluster Surveys (MICS) led by UNICEF [2]. These surveys provide national FGM/C estimates by sampling households that are representative of the national population and asking them a series of questions about FGM/C, such as whether and how the procedure was conducted, at what age, and by type of practitioner.

UNICEF’s most recent estimates report that the number of women and girls that have undergone FGM/C globally have reached 200 million [3]. However, their estimates lack data from countries where the traditional practice is carried out but no data exists (e.g. Saudi Arabia, India, etc.). Additionally, this estimate does not include data from high-income countries where first or following generations of women and girls with FGM/C live [4]. The real prevalence and incidence of FGM/C is unknown in Switzerland and many parts of Europe, as there are no representative surveys similar to DHS or MICS for European countries. Demographers predict that migrants coming from FGM/C-practicing countries towards high income and European countries such as Switzerland will continue to increase [5]. A study published in 2016 analyzing data from the 2011 European census, estimated that, of the 1,353,970 women and girls in Europe aged 10 years and above coming from 1 of the 30 high-prevalence FGM/C countries, an estimated 578,068 women and girls have undergone some type of FGM/C [4]. As of February 2020, there are now 31 FGM/C high-prevalence countries with MICS/DHS estimates, with the addition of the Maldives. The surge of migrants from these countries thus affects the number of women and girls living with FGM/C in Europe. In 2016, estimates in Switzerland showed that around 14,700 women and girls were respectively living with FGM/C or were considered to be theoretically at risk of having undergone or undergoing FGM/C in the future due to their geographical origin only [6]. Since then, estimates have not been updated.

Maria Roth Bernasconi’s parliament initiative to introduce a specific Swiss penal code article against female genital mutilation was the catalyst for the Swiss government’s involvement in the issue of FGM/C [7]. The Federal Office of Public Health (FOPH) has been funding awareness-raising and prevention measures aimed at preventing FGM/C through the national program Migration and Health since 2003. The State Secretariat for Migration, SEM, has been involved in these activities since 2010 as well. In 2015, the National Council decided to support a network to tackle female genital mutilation for the 2016–2019 period. This period has been prolonged until 2021 [8].

More recent and regular estimates on women and girls affected by FGM/C need to be carried out in Switzerland to guide policies. One of the biggest challenges to getting up-to-date estimates is to determine a reliable number of migrant women and girls by country of origin, which usually requires access to census data of the country—limiting estimates to ten-year periods for many European countries. Switzerland is in a unique position because since 2010, the Swiss census is carried out annually, providing a modern statistical database for researchers, policy makers, etc. to observe various data points on a continuous basis [9].

The aim of this study is to update the indirect prevalence estimates for women and girls living with FGM/C in Switzerland, using data from the Swiss Federal Statistical Office of migrant women and girls, born in one of the 30 high-prevalence FGM/C countries that are currently living in Switzerland, i.e. first-generation migrants. Such an update is the first step of a wider research project conducted in Switzerland in 2018 entitled “Female Genital Mutilation/Cutting with a focus on prevalence, risk factors and Swiss health care professionals’ capacities”.

## Methods

We used a similar methodology to Yoder and Van Baelen [4, 10], applying FGM/C DHS and MICS prevalence figures (for girls and women age 15–49) from high-prevalence countries to the number of migrant women and girls living in Switzerland.

We applied the total country prevalence estimates of women aged 15–49 to all migrant women and girls living in Switzerland from the same countries. We also conducted a separate analysis for girls aged 0–14, where we applied the prevalence estimates of girls 0–14 to all migrant girls 0–14 living in Switzerland from the same countries. Where no prevalence estimates for girls 0–14 were available, we applied the prevalence estimates for girls 15–19.

We used the most recent MICS or DHS estimates available for each year (Table 1) and multiplied them to the number of immigrant women and girls from each FGM/C high-prevalence country from 2010 to 2018 based on the Swiss Federal Statistical Office (FSO)‘s Interactive Database. USAID’s Demographic and Health Surveys (DHS) and UNICEF’s Multiple Indicator Cluster Surveys (MICS) are large-scale population-based surveys that produce estimates of socioeconomic and health indicators in low- and middle-income countries [1, 2]. The DHS and MICS surveys have played an important role in the reporting of FGM/C prevalence data in low- and middle- income countries over the past 30 years. Table 1 shows the prevalence estimates from women and girls aged 15–49 that are available for 30 countries where FGM/C is practiced that we used in our study.

**Table 1:** FGM/C Country Prevalence Estimates

Country	Prevalence Estimate (Women ages 15–49), Source, Year of Publication
Benin	(.129 DHS 2006) (.073 DHS 2011–2012) (.092 MICS 2014)
Burkina Faso	(.758 DHS 2010)
Cameroon	(.014 DHS 2004)
Central African Republic	(.242 MICS 2010)
Chad	(.442 MICS 2010) (.384 DHS 2014–2015)
Djibouti	(.931 MICS 2006)
Egypt	(.911 DHS 2008) (.923 DHS 2014) (.872 DHS 2015)
Eritrea	(.83 PHS 2010)
Ethiopia	(.743 DHS 2005) (.652 DHS 2016)
Gambia	(.763 MICS 2010) (.749 DHS 2013) (.757 MICS 2018)
Ghana	(.038 MICS 2006) (.038 MICS 2011)
Guinea	(.956 DHS 2005) (.969 DHS/MICS 2012) (.968 MICS 2016) (.945 DHS 2018)
Guinea-Bissau	(.498 MICS 2010) (.449 MICS 2014)
Iraq	(.081 MICS 2011) (.074 MICS 2018)
Ivory Coast	(.364 MICS 2006) (.382 DHS 2011–2012) (.367 MICS 2016)
Kenya	(.271 DHS 2008–2009) (.21 DHS 2014)
Liberia	(.582 DHS 2007) (.498 DHS 2013)
Mali	(.885 MICS 2010) (.914 DHS 2013) (.827 MICS 2015) (.886 DHS 2018)
Mauritania	(.722 MICS 2007) (.694 MICS 2011) (.666 MICS 2015)
Niger	(.022 DHS/MICS 2006) (.02 DHS/MICS 2012)
Nigeria	(.296 DHS 2008) (.27 MICS 2011) (.248 DHS 2013) (.184 MICS 2016–2017) (.195 DHS 2018)
Senegal	(.257 DHS/MICS 2010–2011) (.247 DHS 2014) (.242 DHS 2015) (.227 DHS 2016) (.24 DHS 2017)
Sierra Leone	(.88 MICS 2010) (.896 DHS 2013) (.861 MICS 2017)
Somalia	(.979 MICS 2006)
Sudan & South Sudan	(.876 SHHS 2010) (.866 MICS 2014)
Tanzania	(.146 DHS 2010) (.1 DHS 2015–2016)
Togo	(.039 MICS 2010) (.047 DHS 2013–2014)
Uganda	(.006 DHS 2006) (.014 DHS 2011) (.03 DHS 2016)
Yemen	(.215 FHS 2003) (.185 DHS 2013)

We used the Swiss Federal Statistical Office’s (FSO) publicly available interactive database STAT-TAB to obtain the number of female permanent and non-permanent residents living in Switzerland from 2010 to 2018 from high FGM/C prevalence countries [11].

We included women and girls of all ages, who have a residence permit labeled “Swiss”, Residence permit (permit B), a settlement permit (permit C), a residence permit with gainful employment (permit Ci), a status of provisionally admitted person (permit F), or of asylum seeker (permit N), or who were diplomats, international civil servants with diplomatic immunity and international civil servants without diplomatic immunity. The citizenship countries that we included were the ones for which FGM/C estimates were available and is known to be practiced: Benin, Burkina Faso, Cameroon, Central African Republic, Chad, Ivory Coast, Djibouti, Egypt, Eritrea, Ethiopia, Gambia, Ghana, Guinea, Guinea-Bissau, Kenya, Liberia, Mali, Mauritania, Niger, Nigeria, Senegal, Sierra Leone, Somalia, Sudan and South Sudan, Tanzania, Togo, Uganda, Iraq, Yemen (Table 2) [3]. Table 2 shows the number of migrant women and girls of all ages living in Switzerland from high FGM/C prevalence countries between 2010-2018. This differs slightly from the UNICEF Switzerland estimates from 2012, as Zambia has since been excluded, and Iraq has been included [12].

**Table 2:** Migrant Women and Girls in Switzerland 2010–2018

Country	Swiss 2010 Migrants Living in Switzerland [WOMEN & GIRLS]	Swiss 2011 Migrants Living in Switzerland [WOMEN & GIRLS]	Swiss 2012 Migrants Living in Switzerland [WOMEN & GIRLS]	Swiss 2013 Migrants Living in Switzerland [WOMEN & GIRLS]	Swiss 2014 Migrants Living in Switzerland [WOMEN & GIRLS]	Swiss 2015 Migrants Living in Switzerland [WOMEN & GIRLS]	Swiss 2016 Migrants Living in Switzerland [WOMEN & GIRLS]	Swiss 2017 Migrants Living in Switzerland [WOMEN & GIRLS]	Swiss 2018 Migrants Living in Switzerland [WOMEN & GIRLS]
Benin	116	124	147	156	145	150	146	143	127
Burkina Faso	168	180	181	180	187	189	189	199	192
Cameroon	2694	2724	2721	2728	2724	2741	2760	2737	2705
Central African Republic	28	27	31	24	25	24	29	33	33
Chad	59	57	62	73	71	70	68	64	64
Djibouti	11	13	14	14	15	18	19	16	16
Egypt	668	736	761	813	826	827	834	865	864
Eritrea	3558	5017	7321	8388	10,300	12,859	14,339	15,600	16,543
Ethiopia	1495	1535	1668	1772	1847	1946	2050	2076	2095
Gambia	64	68	86	98	106	123	121	130	141
Ghana	693	688	701	707	715	708	714	715	723
Guinea	215	229	260	269	287	299	319	335	362
Guinea-Bissau	19	34	45	49	47	54	53	54	61
Iraq	2768	2821	2794	2809	2901	3490	3719	3725	3804
Ivory Coast	885	921	958	976	984	978	971	1006	1008
Kenya	867	907	922	934	972	999	1023	1028	1072
Liberia	64	66	69	69	63	62	60	59	56
Mali	106	103	115	117	121	104	108	108	115
Mauritania	30	35	35	31	31	28	27	33	32
Niger	41	39	40	42	44	48	42	41	49
Nigeria	695	795	880	857	889	900	909	967	987
Senegal	498	522	547	567	591	632	656	675	672
Sierra Leone	79	71	75	68	64	76	71	74	72
Somalia	2266	2396	2648	2705	2819	3031	3243	3275	3290
Sudanb	256	274	289	286	312	324	373	373	409
Tanzania	163	165	162	170	174	177	184	183	188
Togo	568	599	628	649	680	697	699	688	687
Uganda	222	214	219	222	214	204	210	235	253
Yemen	210	225	239	250	271	277	266	270	278
Total	19,506	21,585	24,618	26,023	28,425	32,035	34,202	35,707	36,898

Table 3 shows the number of migrant girls aged 0–14 living in Switzerland from 2010 to 2018 from high FGM/C prevalence countries. We used the STAT-TAB database to obtain the information.

**Table 3:** Migrant Girls in Switzerland 2010–2018

Country	Swiss 2010 Migrants Living in Switzerland [GIRLS 0–14]	Swiss 2011 Migrants Living in Switzerland [GIRLS 0–14]	Swiss 2012 Migrants Living in Switzerland [GIRLS 0–14]	Swiss 2013 Migrants Living in Switzerland [GIRLS 0–14]	Swiss 2014 Migrants Living in Switzerland [GIRLS 0–14]	Swiss 2015 Migrants Living in Switzerland [GIRLS 0–14]	Swiss 2016 Migrants Living in Switzerland [GIRLS 0–14]	Swiss 2017 Migrants Living in Switzerland [GIRLS 0–14]	Swiss 2018 Migrants Living in Switzerland [GIRLS 0–14]
Benin	29	32	41	46	36	41	40	41	31
Burkina Faso	27	24	27	20	23	21	24	26	28
Cameroon	409	407	410	413	407	419	425	406	392
Central African Republic	3	3	4	0	0	0	3	4	4
Chad	15	16	20	22	26	29	27	25	24
Djibouti	3	4	5	5	6	7	5	2	2
Egypt	166	178	184	212	216	213	202	207	194
Eritrea	1084	1556	2214	2685	3235	3923	4632	5355	5970
Ethiopia	367	360	412	417	418	435	465	496	497
Gambia	10	12	20	26	27	34	31	33	39
Ghana	150	144	150	156	154	150	142	138	133
Guinea	48	49	63	62	64	80	87	89	102
Guinea-Bissau	4	7	11	11	11	12	13	13	16
Iraq	934	918	885	895	930	1152	1233	1211	1253
Ivory Coast	120	135	147	160	159	158	157	167	163
Kenya	94	115	118	117	139	134	143	136	141
Liberia	15	16	19	18	15	15	12	12	13
Mali	22	20	23	22	20	15	20	19	16
Mauritania	5	10	9	6	5	3	4	8	6
Niger	8	10	10	11	12	13	10	9	11
Nigeria	172	210	225	227	228	242	240	251	260
Senegal	75	78	85	97	107	116	121	123	119
Sierra Leone	18	19	20	19	19	22	20	21	17
Somalia	754	817	925	973	1005	1038	1092	1151	1172
Sudanb	77	87	92	83	89	84	100	93	103
Tanzania	22	23	22	23	21	22	27	26	30
Togo	158	175	187	187	201	205	198	185	180
Uganda	36	28	31	34	31	24	25	31	41
Yemen	75	77	77	84	89	85	70	67	65
Total	4900	5530	6436	7031	7693	8692	9568	10,345	11,022

## Results

Table 4 describes the estimated total number of women and girls in Switzerland that are living with FGM/C. Indonesia was excluded because there are no DHS and MICS prevalence estimates for the country. In 2010, there were 914 women and girls from Indonesia living in Switzerland, and in 2018, there were 1229.

**Table 4:** Applied Indirect Estimates for Women and Girls (Age 15+) 2010–2018

Country	Swiss 2010 Applied Indirect Estimate [WOMEN & GIRLS]	Swiss 2011 Applied Indirect Estimate [WOMEN & GIRLS]	Swiss 2012 Applied Indirect Estimate [WOMEN & GIRLS]	Swiss 2013 Applied Indirect Estimate [WOMEN & GIRLS]	Swiss 2014 Applied Indirect Estimate [WOMEN & GIRLS]	Swiss 2015 Applied Indirect Estimate [WOMEN & GIRLS]	Swiss 2016 Applied Indirect Estimate [WOMEN & GIRLS]	Swiss 2017 Applied Indirect Estimate [WOMEN & GIRLS]	Swiss 2018 Applied Indirect Estimate [WOMEN & GIRLS]
Benin	14.964	9.052	10.731	11.388	13.340	13.800	13.432	13.156	11.684
Burkina Faso	127.344	136.440	137.198	136.440	141.746	143.262	143.262	150.842	145.536
Cameroon	37.716	38.136	38.094	38.192	38.136	38.374	38.640	38.318	37.870
Central African Republic	6.776	6.534	7.502	5.808	6.050	5.808	7.018	7.986	7.986
Chad	26.078	25.194	27.404	32.266	27.264	26.880	26.112	24.576	24.576
Djibouti	10.241	12.103	13.034	13.034	13.965	16.758	17.689	14.896	14.896
Egypt	608.548	670.496	693.271	740.643	762.398	721.144	727.248	754.280	753.408
Eritrea	2953.140	4164.110	6076.430	6962.040	8549.000	10,672.970	11,901.370	12,948.000	13,730.690
Ethiopia	1110.785	1140.505	1239.324	1316.596	1372.321	1445.878	1336.600	1353.552	1365.940
Gambia	48.832	51.884	65.618	73.402	79.394	92.127	90.629	97.370	106.737
Ghana	26.334	26.144	26.638	26.866	27.170	26.904	27.132	27.170	27.474
Guinea	205.540	218.924	251.940	260.661	278.103	289.731	308.792	324.280	342.090
Guinea-Bissau	9.462	16.932	22.410	24.402	21.103	24.246	23.797	24.246	27.389
Iraq	224.210	228.501	226.314	227.529	234.981	282.690	301.239	301.725	281.496
Ivory Coast	322.140	351.822	365.956	372.832	375.888	373.596	356.357	369.202	369.936
Kenya	234.957	245.797	249.862	253.114	204.120	209.790	214.830	215.880	225.120
Liberia	37.248	38.412	40.158	34.362	31.374	30.876	29.880	29.382	27.888
Mali	93.810	91.155	101.775	106.938	110.594	86.008	89.316	89.316	101.890
Mauritania	21.660	24.290	24.290	21.514	21.514	18.648	17.982	21.978	8.986
Niger	0.902	0.858	0.800	0.840	0.880	0.960	0.840	0.820	0.980
Nigeria	205.720	214.650	237.600	212.536	220.472	223.200	167.256	177.928	192.465
Senegal	127.986	134.154	140.579	145.719	145.977	152.944	148.912	162.000	161.280
Sierra Leone	69.520	62.480	66.000	60.928	57.344	68.096	63.616	63.714	61.992
Somalia	2218.414	2345.684	2592.392	2648.195	2759.801	2967.349	3174.897	3206.225	3220.910
Sudanb	224.256	240.024	253.164	250.536	270.192	280.584	323.018	323.018	354.194
Tanzania	23.798	24.090	23.652	24.820	25.404	17.700	18.400	18.300	18.800
Togo	22.152	23.361	24.492	30.503	31.960	32.759	32.853	32.336	32.289
Uganda	1.332	2.996	3.066	3.108	2.996	2.856	0.630	0.705	0.759
Yemen	45.150	48.375	51.385	46.250	50.135	51.245	49.210	49.950	51.430
Total	9059.01	10,593.10	13,011.079	14,081.462	15,873.622	18,317.183	19,650.957	20,841.151	21,706.691

The evolution of migratory flows throughout the past years has had an effect on the total number of female migrants from these high-prevalence FGM/C countries (Table 2). Between 2010 and 2018, the total number of female migrants has increased, particularly from Eritrea (5-fold increase), with smaller increases from Ethiopia, Egypt, Gambia, Iraq, Kenya, Nigeria, Senegal, Somalia, and Sudan & South Sudan. The number of women from Chad, Sierra Leone and Liberia has slightly declined, while others have stayed stable. Thus, the number of girls and women affected by FGM/C has changed as well.

Over the past decade, there have been significant increases in the number of estimated women and girls living with FGM/C in Switzerland. Our estimates show that in 2010, of the 19,506 women and girls living in Switzerland coming from 1 of the 30 countries where FGM/C is traditional, an estimated 9059 were subjected to the harmful practice. In 2018, of the 36,898 women and girls living in Switzerland coming from 1 of the 30 high prevalence FGM/C countries, an estimated 21,706 have been subjected to the harmful practice.

More than 16,000 of the 36,898 migrant women from the FGM/C high prevalence countries in 2018 come from Eritrea. The indirect estimation of Eritrean women living in Switzerland, where FGM/C estimated prevalence is among the highest in the world, is 13,730. The second highest migrant group of this population comes from Somalia, where the FGM/C estimated prevalence is almost 98%. Out of 3290 women and girls living in Switzerland from Somalia, the applied indirect estimate is 3220 women.

Some countries have made improvements to their prevalence rates over the past years, for example Ethiopia, whose prevalence was .742 in 2010 and has decreased to .650 in 2016, which has an impact on the number of women and girls originating from these countries estimated to be living with FGM/C in Switzerland. In 2010, out of 1495 women and girls from Ethiopia, 1110 were estimated to be living with FGM/C. Fast forward to 2018, 1365 women and girls are estimated to be living with FGM/C out of 2095 total migrant women from Ethiopia.

Table 5 describes the estimated total number of girls ages 0–14 in Switzerland that are at risk of or have undergone FGM/C. This number is based on the indirect prevalence calculation, using data from the Swiss Federal Statistical Office of migrant girls born in one of the 30 high-prevalence FGM/C countries that are currently living in Switzerland (Tables 3 and 4), and multiplied by each country’s most recent DHS and MICS prevalence estimates for ages 0–14. Where estimates for this age group were not available, estimates for the 15–19 age group were used. In 2018, of the 11,022 girls living in Switzerland coming from 1 of the 30 high prevalence FGM/C countries, 3512 are estimated to be at risk or have been subjected to the harmful practice. Migrant girls from countries such as Eritrea, Gambia, Guinea, Senegal and Somalia all saw increases in the number of girls aged 0–14 that are estimated to be living with or at risk of FGM/C in Switzerland between 2010 and 2018. Over the past 10 years, some countries saw a decrease in the number of girls aged 0–14 that are estimated to be living with FGM/C in Switzerland, such as Egypt, Ethiopia, Kenya, Liberia, and Yemen,

**Table 5:** Applied Indirect Estimates for Girls at risk or having undergone FGM/C (Ages 0–14) 2010–2018

Country	Swiss 2010 Applied Indirect Estimate [GIRLS 0–14]	Swiss 2011 Applied Indirect Estimate [GIRLS 0–14]	Swiss 2012 Applied Indirect Estimate [GIRLS 0–14]	Swiss 2013 Applied Indirect Estimate [GIRLS 0–14]	Swiss 2014 Applied Indirect Estimate [GIRLS 0–14]	Swiss 2015 Applied Indirect Estimate [GIRLS 0–14]	Swiss 2016 Applied Indirect Estimate [GIRLS 0–14]	Swiss 2017 Applied Indirect Estimate [GIRLS 0–14]	Swiss 2018 Applied Indirect Estimate [GIRLS 0–14]
Benin	0.06	0.10	0.12	0.14	0.07	0.08	0.08	0.08	0.062
Burkina Faso	3.59	3.19	3.59	2.66	3.06	2.79	3.19	3.46	3.724
Cameroon	2.86	2.85	2.87	2.89	2.85	2.93	2.98	2.84	2.744
Central African Republic	0.02	0.02	0.03	0.00	0.00	0.00	0.02	0.03	0.032
Chad	1.83	1.95	2.44	2.68	2.57	2.87	2.67	2.48	2.376
Djibouti	1.46	1.94	2.43	2.43	2.91	3.40	2.43	0.97	0.97
Egypt	40.01^d^	42.90^d^	44.34^d^	51.09^d^	46.22^d^	30.03	28.48	29.19	27.354
Eritrea	478.04	686.20	976.37	1184.09	1426.64	1730.04	2042.71	2361.56	2632.77
Ethiopia	138.36	135.72	155.32	157.21	157.59	164.00	73.01	77.87	78.029
Gambia	4.24	5.09	8.48	19.84^a^	20.60^a^	25.94^a^	23.65^a^	25.18^a^	19.734
Ghana	2.10^a^	0.58	0.60	0.62	0.62	0.60	0.57	0.55	0.532
Guinea	27.26	27.83	28.67	28.21	29.12	36.40	39.41	40.32	39.882
Guinea-Bissau	1.55	2.71	4.26	4.26	3.26	3.55	3.85	3.85	4.736
Iraq	192.40^b^	189.11	182.31	184.37	191.58	237.31	254.00	249.47	6.265
Ivory Coast	11.40	14.18	15.44	16.80	16.70	16.59	17.11	18.20	17.767
Kenya	13.72^a^	16.79^a^	17.23^a^	17.08^a^	3.89	3.75	4.00	3.81	3.948
Liberia	5.37^a^	5.73^a^	6.80^a^	5.60	4.67	4.67	3.73	3.73	4.043
Mali	16.41	14.92	17.16	15.22	13.84	11.46	15.28	14.52	11.632
Mauritania	3.29	5.48	4.93	3.29	2.74	1.60	2.13	4.26	3.192
Niger	0.07^a^	0.09^a^	0.14^a^	0.15^a^	0.17^a^	0.18^a^	0.14^a^	0.13^a^	0.154^a^
Nigeria	51.43	40.32	43.20	38.36	38.53	40.90	60.72	63.50	49.92
Senegal	9.68^c^	10.06^c^	10.97	12.51	13.80	16.94	16.46	17.22	16.66
Sierra Leone	1.80	1.90	2.00	14.12^a^	14.12^a^	16.35^a^	14.86^a^	1.76	1.428
Somalia	346.84	375.82	425.50	447.58	462.30	477.48	502.32	529.46	539.12
Sudanb	28.49	32.19	34.04	30.71	28.04	26.46	31.50	29.30	32.445
Tanzania	0.75	0.78	0.75	0.78	0.71	1.03	1.27	1.22	1.41
Togo	0.63	0.70	0.75	0.56	0.60	0.62	0.59	0.56	0.54
Uganda	0.18	0.03	0.03	0.03	0.03	0.02	0.03	0.03	0.041
Yemen	16.13	16.555	16.56	13.78^a^	14.60^a^	13.94^a^	11.48^a^	10.99^a^	10.66^a^
Total	1399.97	1635.72	2007.32	2257.07	2501.81	2871.93	3158.67	3496.51	3512.17

^a^Estimates for ages 15–19 were used when estimates for 0–14 were unavailable

^b^In Iraq, estimates for 2011 were used when estimates for 2010 were unavailable

^c^Estimates for Senegal for 2010–2011 are for girls ages 0–10, not 0–15

^d^ Estimates for Egypt from 2010 to 2014 include ages 0–19

The applied indirect estimates for 2018 show a significant decrease for the number of migrant girls with FGM/C from Iraq. New prevalence estimates reported that .005% of girls in Iraq are reported to be at risk or have undergone any form of FGM/C. Out of 1253 girls from Iraq, only 6 girls are estimated to be at risk or have undergone FGM/C. All tables and estimations are available in Additional file 1.

## Discussion

The increase in overall migration from the 30 high-prevalence FGM/C countries should not be overlooked. This increased migrant population leads to an increased estimated FGM/C indirect prevalence [11]. Take for example, the large number of Eritreans present in Switzerland. In 2017, 18,088 people sought asylum in Switzerland [13]. The main country of origin of asylum seekers was Eritrea, with 3375 applications, accounting for over 10% of all applications [13]. Eritreans have been fleeing compulsory military service and dictatorship in their country [14]. However, these asylum applications have continued to fall the past couple of years both in Europe and Switzerland [13]. In 2017, asylum applications were down 33% from 2016—one of the main reasons being that Eritrean arrivals had significantly fallen and that had a direct impact on the number of asylum applications.

Indirect estimation is a systematic and affordable method for estimating the number of women with FGM/C in high-income countries [15–17]. Leye et al. outline that these estimations allow policy makers to look for trends as well as evaluate the impact of prevention programs based on reliable approximations [18]. However, it has methodological limitations and may not reflect the actual FGM/C prevalence among migrants in Switzerland or any community.

There are several demographic characteristics that can influence a woman or girl’s likelihood of having undergone FGM/C that are not taken into account when making indirect estimates. The migrant population in Switzerland may or may not be representative of the population in their country of origin due to socio-economic status, regional origin, religion or ethnicity and therefore may not accurately emulate the prevalence of FGM/C in their home country [19]. For example, we cannot accurately rely on indirect measures for migrants from countries where FGM/C prevalence differs greatly according to ethnicity, without taking into account the migrant’s ethnicity, which is often not included in demographic or census data [20, 21].

Indirect estimates do not account for factors that may influence migrant’s change of behavior, attitudes and beliefs towards FGM/C such as laws prohibiting the practice of FGM/C as well as social pressure not to carry out the traditional practice. However, laws do not always explain the diminishing trend of  FGM/C, as similar trends are observed in countries with and without legislation forbidding the practice [22]. The longer migrants stay in Switzerland, the more acculturation is likely to occur, which could lead either to the abandonment of the practice, or to the preservation of the tradition [23].

Prevalence estimations do not account for the many women and girls that are unaware if they underwent the cutting or of the type of FGM/C they may be living with because there is no physical examination of the genitalia, and therefore the estimations of the prevalence in their country of origin may be underreported [24]. Because of that, surveying samples of migrants might inform future estimates and inform interventions, but would also have limitations.

The real prevalence and incidence of FGM/C and the number of minors at risk remain unknown in many countries, including Switzerland. Our estimates look at major age groupings of girls 0–14 and women over 15. We did not take into account age-adjusted groupings by 5-year groups. Additionally, prevalence estimates that would look at both the Swiss region and canton would allow us to implement more targeted interventions. To obtain more accurate indirect estimates, more detailed information on migrant’s ethnicity as well as their region of origin would need to be recorded upon entry into Switzerland, as FGM/C prevalence often varies significantly in certain ethnic groups and regions.

Despite the various limitations to using indirect measures, we can nevertheless show that there has been a significant increase in the number of women and girls living with or at risk of FGM/C in Switzerland since the previous estimates were conducted. Although we must improve our future estimates, our data show that we must also improve the Swiss capacity for FGM/C monitoring, prevention, treatment and training on this population across diverse settings (medical, social, school, asylum, police).

## Conclusion

Our indirect estimates can only partially inform future policy and public health programs. We believe that indirect estimates should be conducted alongside direct estimates. Direct measures may provide more accurate estimates that could guide policy- and clinical decision-making. Surveying samples of migrants to estimate FGM/C prevalence also has limitations, as they might not know whether they experienced FGM/C or be unaware of the type. However, the implementation of questions about history and type of FGM/C could be integrated into routine health examinations for women and girls coming from countries at risk upon entry into Switzerland, as long as the necessary training is provided. We recommend healthcare professionals and medical coders to use our proposed list of codes from the International Classification of Diseases (ICD) to document and code FGM/C, its associated procedures, and complications, as well as girls “at risk” [25]. Because the ICD is already used by countries around the world, our proposed methods would be feasible in many countries with the proper training on how to diagnose, classify and document FGM/C correctly. ICD is an existing tool that allows for standardized international comparisons [25]. Additionally, we hypothesize that accurate documentation and coding of FGM/C by care-givers will provide more reliable data than those obtained through self-reporting. Furthermore, hospital data represents an opportunity to study access and quality of care for patients who underwent FGM/C, providing guidance for health interventions.

These estimates are meant to be compared with direct data obtained from Swiss University Hospitals in the next steps of a wider research project conducted in Switzerland in 2018 entitled “Female Genital Mutilation/Cutting with a focus on prevalence, risk factors and Swiss health care professionals’ capacities. As a follow-up to these estimates, we conducted a study to assess the coded diagnoses of FGM/C in the five Swiss University Hospitals (paper under review).

## Supplementary Information

**Additional file 1.** This is the full excel file of all FGM/C indirect estimates from 2010 to 2018 for women and girls living in Switzerland.

## Data Availability

The datasets generated and/or analysed during the current study are available in the Swiss Statistical Office’ interactive database, STAT-TAB available at: https://www.bfs.admin.ch/bfs/en/home/services/recherche/stat-tab-online-data-search.html The DHS and MICS data used in this paper are publicly available on the respective websites (www.dhsprogram.com; www.mics.unicef.org/surveys). The datasets used and/or analysed during the current study are available from the corresponding author on reasonable request.

## Abbreviations

DHS: Demographic and Health Survey
FGM/C: Female Genital Mutilation/Cutting
MICS: Multiple Indicator Cluster Survey
